# Strangulated Hernia

**Published:** 1869-01

**Authors:** O. C. Gibbs

**Affiliations:** Frewsbury, Chautauqua County, N. Y.


ART. III.—*Strangulated Hernia.* BY O. C. GIBBS, M. D., *Frewsbury, Chautauqua county, N. Y.*

There are but few difficulties more common than hernia, and but few more painful and dangerous to life, when strangulated. Among the adult male population, I think it is safe to say that at least one in ten is afflicted with hernia. I have been in active practice for twenty years, and have never been compelled to use the knife, for the relief of strangulated hernia. Every surgeon knows that the knife is an extremely dangerous expedient in such cases. I perhaps cannot better illustrate my plan of procedure, than to report a case.

January 5th, 1858, was called about 3 o'clock A. M. to see Mr. Knap, twelve miles away. The case was reported to me, as one of strangulated hernia, of forty-eight hours duration, and I was requested to take my instruments, preparatory to an operation. I did as requested, and arrived there about daylight in the morning. As I entered the house, with my case of instruments, the wife and daughter burst out loudly, weeping as though they expected the husband and father was about to be murdered. The history of the case was as follows: The patient had been, for several years, afflicted with hernia, *oblique inguinal;* while at work in the timber woods it had become strangulated. An Eclectic physician had been sent for, and he had labored for twenty-four hours in an ineffectual attempt to reduce it. Failing in this attempt he had ordered a drastic cathartic, to be repeated until the desired operation was secured, and he then left the patient. The result was persistent vomiting, and augmentation of suffering, without accomplishing a cathartic action. At this point a regular physician was sent for, and he attempted its reduction, laboring diligently and long, but without success. He then gave a full dose of morphine, directing them to send for me, with the design of having an operation performed.

At my arrival, breakfast was ready, and I was invited to eat before operating. I went to the bedside of my patient, forced the forefinger of my right hand up the inguinal canal, and crowded the first joint through the stricture, and then used the full force of my right arm in stretching or lacerating the stricture. Time occupied not exceeding two minutes. I then sat down to my breakfast without making the least effort at reduction. The result was a free gush of wind from the bowels, and an urgent demand to get up and use the chamber vessel, which he did effectually, within less than five minutes from the time I dilated the stricture. The hernia had reduced itself, the bowels moved freely, and the patient was out of danger and at perfect ease, within less than ten minutes from the time of my first seeing him.

As before remarked, in twenty years I have never seen a case but what this method of treatment would prove successful, and I believe in ninety-nine cases in a hundred the pain and risk of a knife can be dispensed with.

